# Intratumoral Chemotherapy: The Effects of Drug Concentration and Dose Apportioning on Tumor Cell Injury

**DOI:** 10.3390/bioengineering11080809

**Published:** 2024-08-09

**Authors:** Jacob S. Warner, C. Matthew Kinsey, Jason H. T. Bates, Vitor Mori

**Affiliations:** Division of Pulmonary & Critical Care Medicine, Department of Medicine, University of Vermont College of Medicine, Burlington, VT 05405, USA

**Keywords:** non-small cell lung cancer, etoposide, cisplatin, doxorubicin, intratumoral injection, endobronchial ultrasound

## Abstract

The addition of intravenous (i.v.) chemotherapy to i.v. immunotherapy for patients with lung cancer results in improved overall survival but is limited by synergistic side effects and an unknown, highly variable final cytotoxic dose within the tumor. The synergy between i.v. chemo- and immunotherapies is hypothesized to occur as a result of cell injury caused by chemotherapy, a mechanism demonstrated to drive antigen presentation within the tumor microenvironment. Intratumoral delivery of chemotherapy may thus be optimized to maximize tumor cell injury. To assess the balance between the damage versus the death of tumor cells, we developed a computational model of intratumoral dynamics within a lung cancer tumor for three different chemotherapy agents following direct injection as a function of location and number of injection sites. We based the model on the morphology of a lung tumor obtained from a thoracic CT scan. We found no meaningful difference in the extent of tumor cell damage between a centrally injected versus peripherally injected agent, but there were significant differences between a single injection versus when the total dose was apportioned between multiple injection sites. Importantly, we also found that the standard chemotherapeutic concentrations used for intravenous administration were effective at causing cell death but were too high to generate significant cell injury. This suggests that to induce maximal tumor cell injury, the optimal concentration should be several orders of magnitude lower than those typically used for intravenous therapy.

## 1. Introduction

Lung cancer is the most lethal cancer in both men and women in the United States, representing a fifth of all cancer-related deaths [1,2]. Intravenous (i.v.) chemotherapy doses have historically been determined through dose-ranging studies designed to identify the maximum tolerated dose (e.g., the dose immediately below that which generates significant systemic toxicity) [3,4]. Direct intratumoral delivery minimizes systemic concentrations, allowing for increased concentration inside the tumor but potentially increasing the risk for development of untoward effects within the tumor microenvironment (TME) well before reaching a dose that generates systemic side effects. Intratumoral dosing has not been thoroughly explored in clinical trials, in part due to the complexity of these studies, and is a problem well suited to computational approaches to provide initial estimates and inform dose selection for clinical evaluation. For example, our group previously leveraged computational modeling to show that tumor cell killing is achieved much more effectively by apportioning a given intratumoral dose between multiple injection sites throughout the tumor as opposed to delivering the entire dose in a single central location. We used these data to guide our clinical trial design [5,6].

Intratumoral delivery of chemotherapeutics, most notably cisplatin, has been shown to be effective at killing cancer cells and to be safe in small controlled Phase 1 studies, and is being investigated as a combination therapy with systemic immunotherapy [7,8]. However, intratumoral delivery has the potential to generate high concentrations within the tumor [9], which can result predominately in tumor cell death via apoptosis rather than non-lethal cell damage. This limits the release of the tumor antigens necessary to induce an immune response against the tumor cells [10]. Failure to generate sufficient antigen presentation from cellular injury is well documented in the radiation literature and is one of the reasons why there are no recommended concurrent radiation and immunotherapy treatment protocols for lung cancer [11]. It is thus crucial to determine how to optimize dose and delivery of intratumoral chemotherapeutic agents to maximize tumor cell injury.

We address this problem in the present study by expanding our prior computational model of intratumoral cisplatin transport [5,6] to account for drug-induced tumor cell damage. We focused specifically on the roles of agent concentration and intratumoral injection strategy in maximizing tumor cell injury.

## 2. Methods

### 2.1. Model Development

Our computational model assigned morphology and volume to tumors by segmenting the boundary of the lung tumor from a high-resolution CT scan of the chest, and the tumor volume ($V_{t}$) was 27.7 mL. Each voxel in the tumor model (0.799 × 0.799 × 0.45 mm) is assumed to represent co-localized extracellular and intracellular spaces. We represent an injected agent as initially forming a pocket in the tumor by pushing tumor tissue away from the injection site. The drug pocket is considered to be a single well-mixed compartment with the same volume of the injected drug $\left(V_{i}\right)$ with a spherical morphology, defined by a surface Ω and area $A_{i}$. $\varphi_{p}(t)$ is the concentration of the drug in the pocket compartment at time t. The initial condition of the pocket compartment is given by:

(1) $$\varphi_{p}\left(t=0\right)=\varphi_{0}$$

where $\varphi_{0}$ is the injected drug concentration. The agent diffuses through Ω to the neighboring voxels of the extracellular space with a diffusion coefficient $D_{p}$, following Fick’s first law of diffusion:

(2) $$\frac{d\varphi_{p}(t)}{dt}=-\frac{A_{i}}{V_{i}}\left[-D_{p}\;\nabla \varphi_{e}\left(\overset{⃑}{r}\in \Omega ,t\right)\right]$$

where $\varphi_{e}(\overset{⃑}{r},t)$ is the extracellular concentration at time t in the voxel located at position $\overset{⃑}{r}$.

To fulfill continuity at the interface between the extracellular space and the pocket compartment, we have

(3) $$\varphi_{p}\left(t\right)=\varphi_{e}\left(\overset{⃑}{r}\in \Omega ,t\right).$$

Upon reaching the extracellular space, the drug (1) diffuses throughout the extracellular space with a spatially uniform diffusion constant D, (2) is irreversibly cleared by the co-localized tumor perfusion into a well-mixed fluid compartment of volume $V_{f}=12.2\;L$ at a constant rate $k_{f}$, and (3) is taken up irreversibly into the co-localized intracellular space with rate constant $k_{i}$. $\varphi_{i}(\overset{⃑}{r},t)$ is the extracellular concentration at time t in the voxel located in the position $\overset{⃑}{r},$ and $\varphi_{f}(t)$ is the concentration of the drug in the fluid compartment at time t. Processes 1, 2, and 3 are described by Equations (4), (5), and (6), respectively:

(4) $$\frac{d\varphi_{e}(\overset{⃑}{r},t)}{dt}=D\nabla^{2}\varphi_{e}\left(\overset{⃑}{r},t\right)-(k_{i}+k_{f})\varphi_{e}(\overset{⃑}{r},t)$$

Boundary conditions are based on the tumor morphology determined from the segmented CT scan. We assume that the tumor capsule prevents any injected agent from leaking out via the tumor boundary and is instead reflected back into the tumor. Therefore, leakage through the vasculature is the only pathway for the drug to leave the tumor.

We let the pocket initially formed by the injected agent alter the shape of the tumor boundary in two different ways. For homogenous expansion, we let the tumor perimeter expand homogenously to accommodate the injected volume. For local expansion, we expanded the tumor along each direction from the injection site to the tumor border by an amount inversely proportional to the distance to the border.

We injected the agent either in its entirely at a single location in the tumor or apportioned it equally between five different sites across the span of the tumor. In the latter case, the drug was injected into each site sequentially in random order. Additionally, if the pocket yielded by two sequential injections overlaps, we assume that they connect, forming a single larger pocket with a total volume given by the sum of the volume of each injection while maintaining the same drug concentration, as each voxel can only hold a finite amount of fluid. We assume for all injection scenarios that each agent was delivered in an aqueous solution having a volume corresponding to 25% of the tumor volume, in line with a previous Phase I clinical trial for intratumoral chemotherapy [8].

Following exposure to the drug, tumor cells are classified into three categories—live, injured, and dead—based on the drug concentration in the local intracellular space. Tumor cell death occurs when the concentration in the intracellular space exceeds a specified lethal threshold, as described previously [5]. Cell injury occurs when intracellular concentration resides between the lethal and sub-lethal injurious thresholds. If the drug concentration in the intracellular space is below the injury threshold, the cell remains unaffected.

We applied this model to the transport of cisplatin, doxorubicin, and etoposide. These agents were selected due to their established activities for treating lung cancer and their capacities to generate markers of immunogenic cell injury, such as inducing calreticulin exposure and the release of HMGB1 and ATP or recruiting effector cells following an injurious but non-lethal dose of the agent [12,13]. Although not included in our model, we assume that cell injury results in the release of these compounds, which then go on to induce an immune response.

### 2.2. Model Parameterization

For simplicity, we assume that $D_{p}=D$, and from now on, refer to both as D. The diffusion constant used for each agent was based on its molecular weight derived using an empirical formula [14]. The values of $k_{i}$ for cisplatin and doxorubicin were obtained from the literature, while $k_{i}$ for etoposide was assumed to be equal to that of doxorubicin given their similar molecular weights. The rate of clearance via tumor perfusion for cisplatin was obtained based on platinum levels in the blood of a patient following intratumoral injection [5,6]. For doxorubicin and etoposide, $k_{f}$ was assumed to be the same as for cisplatin due to their similar molecular weights [15,16].

The threshold lethal drug concentrations in the intracellular compartment were determined from the mean IC50 values for NSCLC from the GDSC [17], which are 1.7 × 10^−5^, 1.3 × 10^−6^, and 5.8 × 10^−5^ M for cisplatin, doxorubicin, and etoposide, respectively. Sub-lethal but injurious concentrations were defined empirically to be within two standard deviations below the mean IC50 value. Hence, minimum injurious concentrations are 1 × 10^−6^, 1.2 × 10^−8^, and 4.5 × 10^−7^ M for cisplatin, doxorubicin, and etoposide. These values align with published values for chemotherapy-induced DAMP release for doxorubicin (1 × 10^−8^–1 × 10^−6^ M) [12], etoposide (2 × 10^−7^–6 × 10^−7^ M) [18], and DNA damage for cisplatin (1 × 10^−6^ M) [19]. Intracellular concentrations below the sub-lethal injurious thresholds were defined as innocuous.

The model parameters for each agent are provided in Table 1:

### 2.3. Model Implementation

The above system of equations was solved numerically with the Finite Difference Method using a timestep of one hour and a 3D grid sampled at the same size as the voxels in the CT scan. The Laplacian in Equation (4) was implemented as the differences between each point and its six neighbors. For each edge voxel, the Laplacian was implemented as the difference between the voxel and the average of however many neighboring voxels corresponded to tumor cells. All coding was performed using Python (version 3.11.5), with plots created using the Matplotlib package [20].

### 2.4. Statistical Tests

Independent *t*-tests were used to compare results from central versus off-center injections. ANOVA was used to compare the results of dose apportioning versus a single injection between and within agents. Tukey’s honest significant difference (HSD) was used to identify pairings that were significantly different if ANOVA was significant. For each agent, the control was the concentration used for intravenous therapy (cisplatin, 1 mg/mL; doxorubicin, 2 mg/mL; etoposide, 20 mg/mL). ANOVA testing was performed using the SciPy library [21]. Significance between pairings tested with Tukey’s HSD was performed with the Statsmodels library [22].

## 3. Results

**Figure 1:**
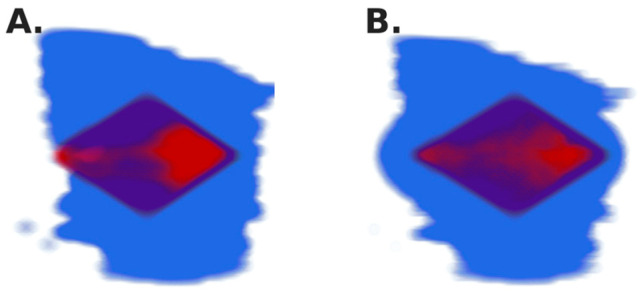
Tumor silhouette (blue) and drug deposition (red) shown with deformation modeled as homogenous expansion (**A**) and local expansion (**B**).

Figure 1 shows visualizations of the tumor immediately following a single, centrally located fluid injection for homogenous tumor expansion (Figure 1A) and local expansion (Figure 1B).

**Figure 2:**
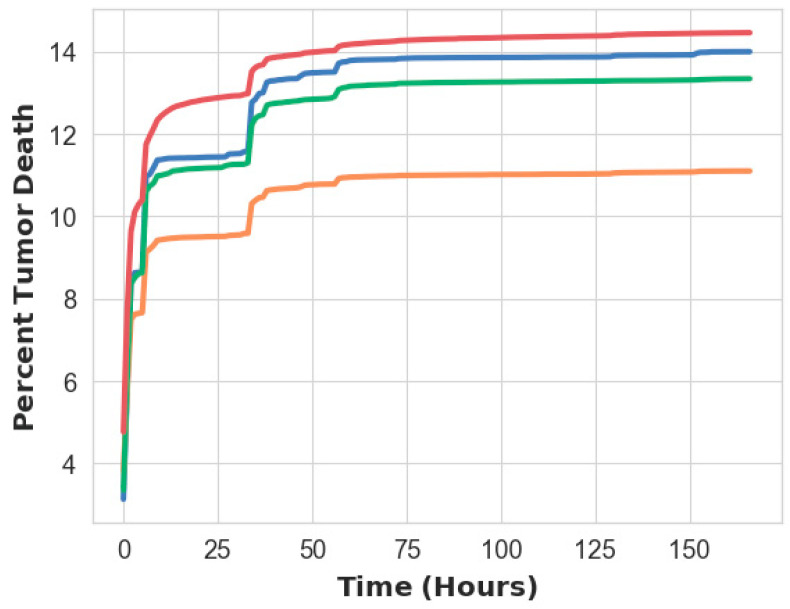
Temporal relation between the % of tumor death over time when the drug is injected into the tumor. Blue line—homogenous tumor expansion, central injection; orange line—homogenous tumor expansion, off-center injection; green line—local expansion, central injection; red line—local expansion, off-center injection.

Figure 2 shows the temporal evolution of the percent tumor volume experiencing a concentration above the lethal threshold in the intracellular space following a single cisplatin injection. The model was simulated over 160 h (~7 days). The greatest cell death was achieved with an off-center injection and local tissue expansion, while the least occurred with an off-center injection and homogenous tissue expansion. A central injection with homogenous and local tissue expansions gave intermediate amounts of cell death. Due to the similarities between the outcomes of the two expansion models, all further simulations were performed using the homogenous tissue expansion model.

**Figure 3:**
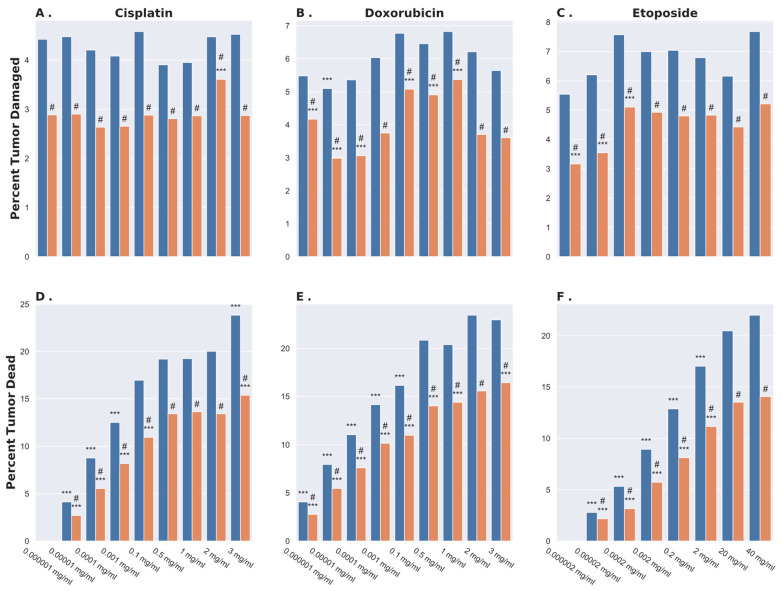
Percent tumor damage (**top** row) and killing (**bottom** row) with cisplatin (**A**,**D**), doxorubicin (**B**,**E**), and etoposide (**C**,**F**) for both dose apportioning (blue) and single (orange) injection strategies. ANOVA testing was performed between doses for each combination of drug and injection strategy using standard intravenous concentrations for each drug as control (1 mg/mL for cisplatin, 2 mg/mL for doxorubicin, and 40 mg/mL for etoposide). Statistical differences are represented by *** (*p* < 0.05). For each combination of drug and dose, differences between single and multiple injections strategies are represented by # (*p* < 0.05).

We compared the relative amounts of tumor cell injury (Figure 3A–C) and tumor cell death (Figure 3D–F) after 14 days of the injection upon exposure to three commonly used chemotherapy agents—cisplatin (Figure 3A,D), doxorubicin (Figure 3B,E), and etoposide (Figure 3C,F). Up to nine different agent concentrations were used (0.000001–3 mg/mL for cisplatin and doxorubicin, and 0.000002–40 mg/mL for etoposide), each in the same total fluid volume, corresponding to 25% of the 27.7 mL tumor volume. We simulated dose apportioning in both a single injection (orange bars) and in five injections (blue bars). For each combination of drug, dose, and apportioning strategy, we performed five simulations with differential initial injection locations that were randomly selected but constrained by the following rules: (1) for a single injection, the needle was placed within a region corresponding to 10% of the tumor volume from the tumor centroid, and (2) for a multiple injection strategy, the individual injections were separated by >1–2 mm.

**Figure 4:**
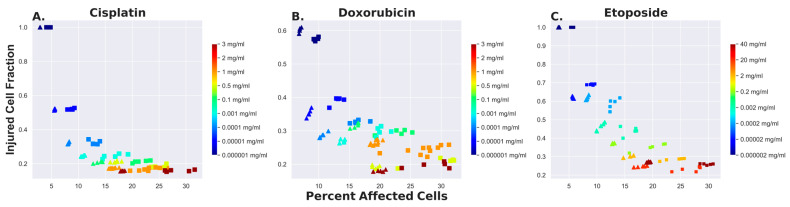
Ratio of tumor fraction damaged by agent to total tumor fraction affected (i.e., either damaged or killed) for cisplatin (**A**), doxorubicin (**B**), and etoposide (**C**). Results obtained from simulating multiple injections are shown by the triangles. Results from a single injection are shown by the squares. The color bar on the right shows the initial drug concentrations.

We determined the injured cell fraction as the ratio of the tumor volume exposed to an injurious level to the total volume of the tumor affected by the drug $\left(\frac{V_{injured}}{V_{injured}+V_{dead}}\right)$. Similarly, the percent affected cells, equivalent to the percentage of the tumor affected by the chemotherapy agent, is defined as $\left(\frac{V_{injured}+V_{dead}}{V_{tumor}}\right)$. Injured cell fraction is plotted versus percent affected cells in Figure 4.

## 4. Discussion

Intratumoral delivery of chemotherapy into lung tumors through bronchoscopy has proven safe in small, well-controlled trials both for early-stage lung cancer [8] and as a salvage therapy for patients without further treatment options [5]. However, studies to date have focused on the effect of the cytotoxic agent on the killing of tumor cells, irrespective of the mechanism of death and potential to augment the immune response. Recent data from multiple studies have altered the paradigm of the use of chemotherapy as an adjuvant to immunotherapy, highlighting the importance of immunogenic cell injury in driving an immune response [23,24,25,26]. This form of cellular injury occurs at lower doses of chemotherapeutic agents and releases immune mediators such as danger associated molecular proteins (DAMPs), whereas the higher doses result in apoptosis and sequestration of the tumor antigens necessary to drive an immune response [10,12].

Najibi et al. [12] showed that several markers of DAMP release, including calreticulin and HMGB-1, as well as multiple immune interaction markers, were increased in tumor cultures with sub-maximal concentrations of doxorubicin. Sriram et al. [10] reported similar findings in mice treated with etoposide, doxorubicin, and mitoxantrone, where both doxorubicin and etoposide increased release at 10 µM, which is in line with the lower concentrations we simulated (1 × 10^−6^–1 × 10^−5^ mg/mL). They also found an increase in dendritic cell-mediated T cell activation, which was in agreement with prior work showing changes in immune cell composition in lung cancers after an intratumoral injection of cisplatin. [9] Our model complements this prior research by suggesting optimum injection strategies to maximize tumor cell injury as well as offering guidance regarding concentration for the commonly used anti-cancer drugs.

Intratumoral approaches have the potential to precisely target the optimal window to maximize tumor cell injury through the ability to achieve specific intratumoral concentrations, less variability in the dose that reaches the tumor, and a more homogenous final intratumoral concentration versus a selected i.v. dose. However, there are little data to inform the selection of the appropriate intratumoral dose and delivery strategy. We targeted this problem by developing a computational model of post-injection drug transport. The main finding of our model is that the intratumoral injection of a chemotherapeutic agent at the concentration given intravenously produces a level of tumor cell injury similar to that of a drug delivered in concentrations 5–6 orders of magnitude lower. However, despite the similar level of tumor cell injury, there is up to 10-fold greater cell death at the standard intravenous concentration. This implies that the typical intravenous concentration comes with a substantial risk of tumor resident immune cell ablation [12]. Hence, dosing for intratumoral injections aimed at immune priming can be optimized by selecting a much lower concentration that is close to the injurious range.

Our model results indicate little effect on treatment outcome due to the way in which a tumor expands during injection or where in the tumor the agent is delivered as a single injection (Figure 2), with just a 2.9% difference in tumor cell death between the simulated scenarios. This is of practical importance because not all tumor regions may be readily accessible for injection, and even the accessible regions may require multiple attempts to ensure central needle placement [8]. As long as the drug is retained entirely within the tumor, flexibility in injection site can reduce the length of the surgical procedure for the patient.

Surprisingly, the different dilutions of the injected agent resulted in minimal variation in the percentage of the tumor receiving sub-lethal injurious doses (Figure 3A–C). As the injected agent diffuses radially away from the site of injection, it forms a sphere centered at the injection site within which the agent concentration is lethal (provided the injected concentration is itself above the lethal concentration). The radius of this lethal sphere depends on the injected agent dose. Immediately beyond the lethal sphere, a concentric shell forms in which the agent concentration is above the cell injury threshold but below the death threshold. The results from our simulations demonstrate that the volume of this shell varies little with different doses. Obviously, if the initial concentration is less than the injury threshold, no cells will be either killed or injured. Conversely, as the initial concentration approaches infinity, all tumor cells will be killed, leaving none merely injured. Therefore, cell injury and cell death are essentially decoupled, allowing injury to be maximized while minimizing the risk of ablating tumor resident immune cells in the tumor.

Figure 4 also captures this dynamic. For all drugs, increasing the drug concentration increases the total volume of the tumor affected by the agent, mainly by killing more cells. Indeed, in the hypothetical limiting case where the concentration tends to infinity, all the tumor cells will reach the cytotoxic threshold, and the injured cell fraction trends toward zero. On the other hand, when the concentration of the injected drug is already below the cytotoxic threshold, cells are only injured rather than killed, in which case the injured cell fraction trends to 1. Moreover, while doxorubicin and etoposide cover a wider range of concentrations with larger values of injured cell fraction (above 0.3), cisplatin tends to flatten out to a horizontal asymptote more quickly (Figure 4). Since diffusion is virtually the same for all three agents, this may reflect the smaller range of injury for cisplatin compared to the other agents. For both doxorubicin and etoposide, the injury threshold is several orders of magnitude lower than the lethal threshold, whereas for cisplatin, the difference is only a factor of ten.

In line with our previous results from a computational model of intratumoral drug delivery aimed at cytotoxicity [5], we found statistically significant benefits of apportioning the delivered dose between multiple injections to increase the tumor volume exposed to a concentration within the sub-lethal injurious range (Figure 3). Our findings thus suggest that maximum cell injury with minimal risk of immune cell ablation occurs when a chemotherapeutic agent is administered in multiple injections at a concentration just above the sub-lethal injurious interval.

There are a number of important limitations to this study, and many are related to simplifying assumptions of the model. For example, we assume that the biophysical properties are uniform throughout the tumor. Moreover, we solely modeled tumor cell injury and death and did not specifically model the subsequent response of immune mediators. This step is necessary, although in many cases not sufficient, for initiating a tumor immune response. Subsequent steps in the immune response require a detailed understanding of the biophysical environment that drives cancer-associated fibroblasts and functionally excludes T cells, the nature of the tumor immune microenvironment (including suppressive and activating T cell subsets and proportions of exhausted, double-negative, and activated T cells), antigen presenting cell density and effectiveness, rates of migration and the extent of extratumoral immune cells, and a number of other factors for which there are currently little data regarding the short term (<1 week) time scale changes induced by these chemotherapeutic agents in human tumors. The sub-lethal injurious interval was selected based on literature values from a number of in vitro studies. Although selecting a different sub-lethal injurious interval would affect the total number of injured cells, our model demonstrated a lack of sensitivity to injury levels by concentration. Finally, we based our computational model on a single tumor morphology, which limits the generalizability of our conclusions.

In conclusion, model simulations suggest that chemotherapy agents delivered at the same concentration as used intravenously generate significant cell death but with little gain in tumor cell injury. The optimal concentration for intratumoral chemotherapy required to maximize tumor cell injury is just above the sub-lethal injurious level, which is orders of magnitude lower than that which is typically used for intravenous therapy. This may offer a substantial benefit to patients through reductions in systemic exposure to the drug and further minimize off-target side effects that are already modest for intratumoral drug delivery.

## Figures and Tables

**Table 1:** Model parameters.

	D (cm^2^/s)	$k_{i}$	$k_{f}\;(/s)$	Lethal Threshold (M)	Injury Threshold (M)	Literature Values DAMP Release (M)
Cisplatin	2.5 × 10^−6^	1.0 × 10^−4^	1.5 × 10^−4^	1.7 × 10^−5^	1 × 10^−6^	1.0 × 10^−6^
Doxorubicin	1.6 × 10^−6^	1.05 × 10^−4^	1.5 × 10^−4^	1.3 × 10^−6^	1.2 × 10^−8^	1.0 × 10^−8^
Etoposide	1.5 × 10^−6^	1.05 × 10^−4^	1.5 × 10^−4^	5.8 × 10^−5^	4.5 × 10^−7^	2.0 × 10^−7^

## Data Availability

The original contributions presented in the study are included in the article, further inquiries can be directed to the corresponding author/s.
